# Three New Phthalide Glycosides from the Rhizomes of *Cnidium officinale* and Their Recovery Effect on Damaged Otic Hair Cells in Zebrafish

**DOI:** 10.3390/molecules26227034

**Published:** 2021-11-21

**Authors:** Hyoung-Geun Kim, Seon Min Oh, Na Woo Kim, Ji Heon Shim, Youn Hee Nam, Trong Nguyen Nguyen, Min-Ho Lee, Dae Young Lee, Tong Ho Kang, Nam-In Baek

**Affiliations:** 1 Graduate School of Biotechnology and Department of Oriental Medicinal Biotechnology, Kyung Hee University, Yongin 17104, Korea; zwang05@khu.ac.kr (H.-G.K.); seonmin88@khu.ac.kr (S.M.O.); nawoonifty@khu.ac.kr (N.W.K.); jee1015235@gmail.com (J.H.S.); 01030084217@hanmail.net (Y.H.N.); ntnguyen@khu.ac.kr (T.N.N.); panjae@khu.ac.kr (T.H.K.); 2 Department of Herbal Crop Research, National Institute of Horticultural and Herbal Science, RDA, Eumseong 27709, Korea; dylee0809@gmail.com; 3 Department of Food Technology and Services, Eulji University, Seongnam 13135, Korea; minho@eulji.ac.kr

**Keywords:** circular dichroism, *Cnidium officinale*, ligusticoside, otic hair cells, phthalide, stereostructure, zebrafish

## Abstract

The extract from *Cnidium officinale* rhizomes was shown in a prior experiment to markedly recover otic hair cells in zebrafish damaged by neomycin. The current study was brought about to identify the principal metabolite. Column chromatography using octadecyl SiO_2_ and SiO_2_ was performed to isolate the major metabolites from the active fraction. The chemical structures were resolved on the basis of spectroscopic data, including NMR, IR, MS, and circular dichroism (CD) data. The isolated phthalide glycosides were assessed for their recovery effect on damaged otic hair cells in neomycin-treated zebrafish. Three new phthalide glycosides were isolated, and their chemical structures, including stereochemical characteristics, were determined. Two glycosides (0.1 μM) showed a recovery effect (*p* < 0.01) on otic hair cells in zebrafish affected by neomycin ototoxicity. Repeated column chromatography led to the isolation of three new phthalide glycosides, named ligusticosides C (**1**), D (**2**), and E (**3**). Ligusticoside C and ligusticoside E recovered damaged otic hair cells in zebrafish.

## 1. Introduction

*Cnidium officinale* Makino (Apiaceae) is a perennial flowering plant extensively cultivated in East Asian countries [1]. The rhizomes of *C. officinale* have been used traditionally in Korea, China, and Japan to treat female sexual disorders such as oligomenorrhea, hypomenorrhea, and amenorrhea by improving blood circulation, but also to relieve pain and inflammation [2,3]. Extracts of *Cnidium officinale* rhizome are reported to have antioxidant [4], anti-inflammatory [5,6], anticancer [5], blood circulation improvement [7], analgesic [6], anticonvulsive [8], and sedative [9] effects. A variety of components were identified as the major metabolites of *Cnidium officinale* rhizome, such as phthalides, phenyl alkanoids, triterpenoids, and polyacetylenes, etc. Among them, phthalide derivatives are the most important components and are reported to be key materials participating in several pharmacological activities of *C. officinale* rhizome [10]. Our advance experiment showed that the *n*-BuOH fraction from *C. officinale* rhizome recovered otic hair cells in zebrafish damaged by neomycin treatment. In a subsequent study presented herein, repeated column chromatography (CC) for the *n*-BuOH fraction yielded three new phthalide glycosides, the chemical structures of which, including stereostructures, were determined without ambiguity based on several spectroscopic data, that is, NMR, IR, MS, and circular dichroism spectroscopy (CD). Only a few glycosides have been isolated from plants. However, the phthalide glycosides with a double bond at C-6 and C-7 in the aglycone moiety have never been reported, to date. The phthalide glycosides were also evaluated for their recovery effect on damaged otic hair cells.

## 2. Results and Discussion

### 2.1. Structure Determination of Three New Phthalide Glycosides

Aqueous EtOH was used to obtain an extract of the rhizomes of *Cnidium officinale*, and the concentrate was fractionated using EtOAc, n-BuOH, and H_2_O. The n-BuOH fraction (Fr) was treated using Sephadex LH-20, silica gel (SiO_2_), and octadecyl SiO_2_ (ODS) as the stationary phase for column chromatography, resulting in the isolation of three new phthalide glycosides. Their chemical structures, including their absolute stereochemistry, were revealed based on MS, IR, NMR, and CD data.

**Figure 1:**
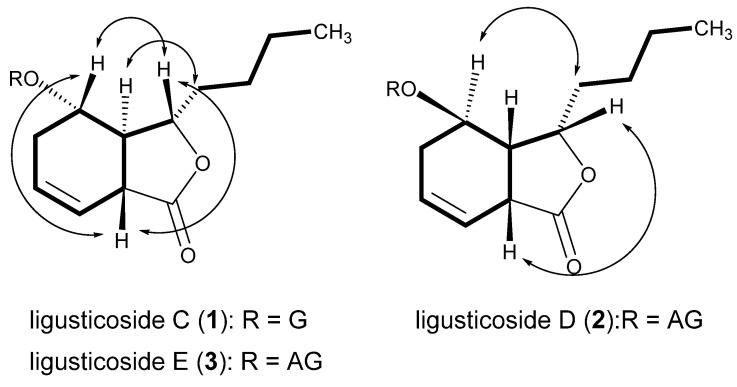
Key correlations in the ^1^H-^1^H COSY (—) and NOESY (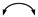 ) spectra. G: *β*-D-glucopyranosyl; AG: *β*-D-apiofuranosyl-(1→6)-*β*-D-glucopyranosyl.

**Figure 2:**
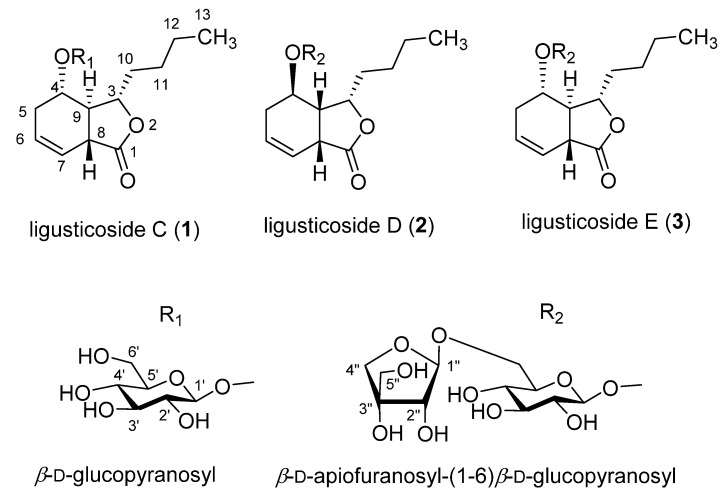
Chemical structures of phthalide glycosides from the rhizome of *Cnidium officinale* Makino.

Compound **1**, a white powder, showed UV absorption at 254 and 365 nm and a deep brown color on TLC upon spraying with 10% sulfuric acid and heating. The molecular formula (MF) was determined to be C_18_H_28_O_8_ (calcd for C_18_H_29_O_8_, 373.1858) from a protonated molecular (PM) at m/z 373.1861 [M + H]^+^ in the high-resolution ESI-MS (HR-ESI-MS). The IR bands showed absorbance bands at 3367 (hydroxyl), 1742 (ester), and 1606 and 1514 cm^−1^ (double bond). In the ^13^C NMR (CMR) spectrum (Table 1), 18 carbon signals, including 6 due to one hexose, were observed. The above data assuredly suggest compound **1** to be a phthalide monoglycoside. The sugar was identified to be a β-glucopyranose from the chemical shifts of the carbon signals due to one hemiacetal (δ_C_ 102.76), four oxygenated methine (δ_C_ 78.06, 78.01, 75.03, 71.85), and one oxygenated methylene (δ_C_ 63.08) moieties. The carbon signals of the aglycone included one ester (δ_C_ 179.04), two olefin methines (δ_C_ 127.66, 122.29), two oxygenated methines (δ_C_ 83.20, 73.19), two methines (δ_C_ 43.90, 42.67), four methylenes (δ_C_ 36.06, 28.77, 27.75, 23.51), and one methyl (δ_C_ 14.31) observed, indicating that the aglycone had a hexahydro-butylphthalide moiety with one hydroxyl group and one double bond. The ^1^H NMR (PMR) spectrum (Table 2; δ_H_, coupling pattern, J in Hz) showed two olefin methines (5.72, br. ddd, 4.2, 6.6, 10.2; 5.71, ddd, 2.4, 4.2, 10.2), two oxygenated methines (4.55, ddd, 5.4, 5.4, 9.6; 4.12, ddd, 3.6, 4.8, 6.6), three methylenes with germinal coupling (2.21, overlapped; 2.19, overlapped; 1.83–1.77, m; 1.60–1.54, m; 1.46–1.39, m; 1.27–1.21, overlapped), one methylene (1.27, overlapped), and one terminal methyl (0.84, t, 7.2), the same as those of the aglycone moiety. The signals of the β-glucopyranose were the same as those of a hemiacetal (4.22, d, 7.8), four oxygenated methines (δ_H_ 3.30 to 3.31), and one oxygenated methylene (3.79, dd, 2.8, 12.0; 3.53, dd, 4.2, 12.0). The ^1^H-^1^H COSY spectrum suggested the connection of carbons with protons shown in Figure 1 The positions of two oxygenated methine carbons (C-3 and C-4) were confirmed from the cross peaks in the HMBC spectrum, that is, H-3 (δ_H_ 4.55)/C-11 (δ_C_ 28.77), C-8 (δ_C_ 42.67), C-4 (δ_C_ 73.19), and C-1 (δ_C_ 179.04); H-4 (δ_H_ 4.12)/C-8, C-3 (δ_C_ 83.20), C-1′ (δ_C_ 102.76), and C-6 (δ_C_ 127.66), respectively. Additionally, the HMBC spectrum confirmed the position of two olefin methines (C-6 and C-7), which showed cross peaks such as H-6 (δ_H_ 5.72)/C-5 (δ_C_ 27.75), C-8, C-4, C-7 (δ_C_ 122.29), and C-1; H-7 (δ_H_ 5.71)/C-5, C-8, C-9 (δ_C_ 43.90), C-6, and C-1, respectively. The sugar was revealed to be linked to the hydroxyl group at C-4 from the cross peaks in the HMBC spectrum as H-1′(δ_H_ 4.22)/C-4 and H-4/C-1′. Consequently, the planar structure was determined as shown in Figure 2. In the NOESY spectrum (Figure 1), H-3 (δ_H_ 4.55) showed a correlation with H-4 (δ_H_ 4.12) and H-8 (δ_H_ 3.27), while H-9 (δ_H_ 2.71) showed a correlation with H-10 (δ_H_ 1.83, 1.60), proving the relative stereostructure as shown in Figure 1 and 2. Compound **1** was hydrolyzed using 1N HCl to give the aglycone, **1a**, which exhibited the positive Cotton effect at 255 nm (Δε 3.22) and the negative Cotton effect at 212 nm (Δε -3.26), indicating the hydroxyl group at C-4 to have an α-position [11,12]. Therefore, the chemical structure, including the absolute stereochemistry, of compound **1** was determined as shown in Figure 2, and it was named ligusticoside C.

Compound **3**, a white powder, showed UV absorption at 254 and 365 nm and a deep brown color on TLC upon spraying with 10% sulfuric acid and heating. MF was determined to be C_23_H_36_O_12_ (calcd for C_23_H_37_O_12_, 505.2281) from a PM at m/z 505.2283 [M + H]^+^ in HR-ESI-MS. The IR bands showed absorbance bands at 3357 (hydroxyl), 1745 (ester), and 1685 and 1458 cm^−1^ (double bond). CMR and PMR (Table 1 and Table 2) were almost the same as those of compound **1** with the exception of the additional signals due to one pentose. The signals of the pentose included those of one hemiacetal (δ_H_ 5.04, d, 2.4, H1”; δ_C_ 111.19, C-1”), one oxygenated quaternary carbon (δ_C_ 80.62, C-3”), one oxygenated methine (δ_H_ 3.89, d, 2.4, H-2”; δ_C_ 78.16, C-2”), and two oxygenated methylene carbons (δ_H_ 3.96, d, 9.6 and δ_H_ 3.76, d, 9.6, H-4”; δ_H_ 3.57, s, H-5”; δ_C_ 75.09, C-4”; δ_C_ 65.57, C-5”). The oxygenated quaternary carbon signal and the singlet oxygenated methylene proton signals suggested the sugar to be a branched-chain aldopentose, which was identified to be an apiofuranose from the chemical shift in CMR. The characteristics of the stereostructure were revealed to be the same as those of compound **1** via the same methods as used previously. Therefore, the chemical structure, including the absolute stereochemistry, of compound **3** was determined as shown in Figure 2, and it was named ligusticoside E.

Compound **2**, a white powder, showed UV absorption at 254 and 365 nm and a deep brown color on TLC upon spraying with 10% sulfuric acid and heating. MF was determined to be C_23_H_36_O_12_ (calcd for C_23_H_37_O_12_, 505.2281) from a PM at m/z 505.2285 [M + H]^+^ in HR-ESI-MS. The IR bands showed absorbance bands at 3356 (hydroxyl), 1747 (ester), and 1600 and 1456 cm^−1^ (double bond). From PMR and CMR, we concluded that the planar structure was the same as that of **3**. In the NOESY spectrum of compound **2** (Figure 1), H-3 (δ_H_ 4.57) showed a correlation with H-8 (δ_H_ 3.60) and H-9 (δ_H_ 2.78–2.74), while H-10 (δ_H_ 2.18–2.12 and δ_H_ 2.05) showed a correlation with not H-4 (δ_H_ 4.15) but H-3, proving the relative stereostructure. The aglycone of compound **2**, **2a**, obtained by acid hydrolysis, exhibited the negative Cotton effect at 250 nm (Δε −1.66) and the positive Cotton effect at 218 nm (Δε 1.29), indicating the n-butyl group at C-3, the hydrogen at C-9, and the hydroxyl group at C-4 to have α-, β-, and β-positions, respectively [12,13]. Therefore, the chemical structure, including the absolute stereochemistry, of compound **2** was determined as shown in Figure 2, and it was named ligusticoside D.

To date, lots of phthalides have been isolated from *Cnidium officinale* or *Angelica gigas*. However, less than 10 phthalide glycoside have been reported. Among them, most glycosides have a double bond at C-7 and C-8 or at C-8 and C-9. Ligusticosides C–E isolated in this study are the first occurrence for phthalide glycosides to have a double bond at C-6 and C-7 in the aglycone moiety.

### 2.2. Recovery Effects for the Extract, Solvent Fractions, and Compounds ***1**–**3*** on Otic Hair Cells in Zebrafish Damaged by Neomycin Treatment 

**Figure 3:**
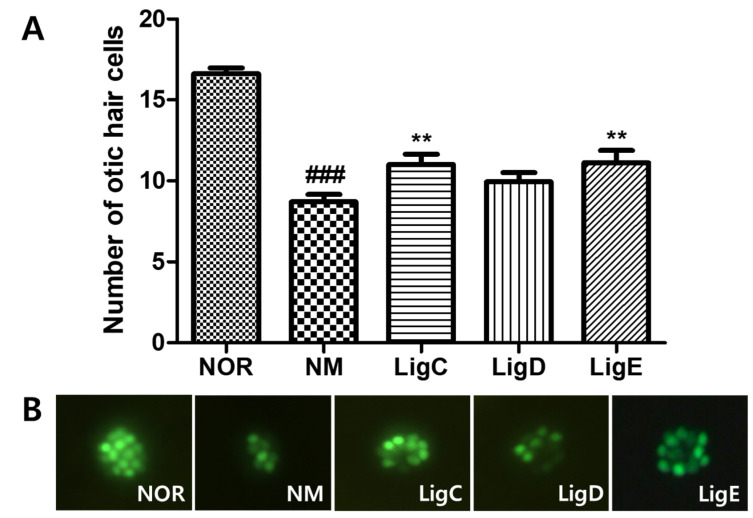
Recovery of otic hair cells after neomycin-induced hair cell damage. (**A**) The numbers of otic hair cells in the untreated group (NOR), the neomycin treatment group (NM), and the phthalide glycoside treatment groups (LigC, LigD, LigE; 0.1 µM). (**B**) Fluorescence images of the zebrafish otic hair cells. Hair cells were stained with 0.1% YO-PRO-1. Data are presented as means ± SEM. ** *p* < 0.01 (control versus treated groups). ### *p* < 0.001 (normal group versus control group). LigC, ligusticoside C (1); LigD, ligusticoside D (2); LigE, ligusticoside E (3).

The recovery effects of the phthalide glycosides **1**–**3** on otic neuromast hair cells exposed to neomycin were evaluated. Treatment with the ototoxic drug severely damaged the hair cells, which significantly decreased in number (*p* < 0.001). Ligusticoside C (**1**) and ligusticoside E (**3**) significantly recovered the damaged hair cells (*p* < 0.01), while ligusticoside D (**2**) exhibited an effect without statistical significance (Figure 3). Therefore, a phthalide glycoside-enriched fraction can be a promising source to prevent or treat the auditory pathological symptoms. 

## 3. Materials and Methods

### 3.1. Plant Materials

The Department of Herbal Crop Research, RDA, Eumseong, Korea, supplied the rhizomes of *Cnidium officinale*, and Dr. J.T. Jeong, Department of Herbal Crop Research, identified them. A voucher specimen (NPCL-20200023) was stored at the Natural Products Chemistry Laboratory of Kyung Hee University, Yongin, Korea.

### 3.2. General Experimental Procedures

The equipment and chemicals used for the isolation of the phthalide glycosides and structure determination of the isolated metabolites were selected by referring to the literature [14]. For the breeding and maintenance of the zebrafish to test the recovery effect on otic hair cells damaged by neomycin treatment, we followed the methods previously reported in the literature [15].

### 3.3. Isolation of Phthalide Glycosides from the Rhizomes of Cnidium officinale

**Figure 4:**
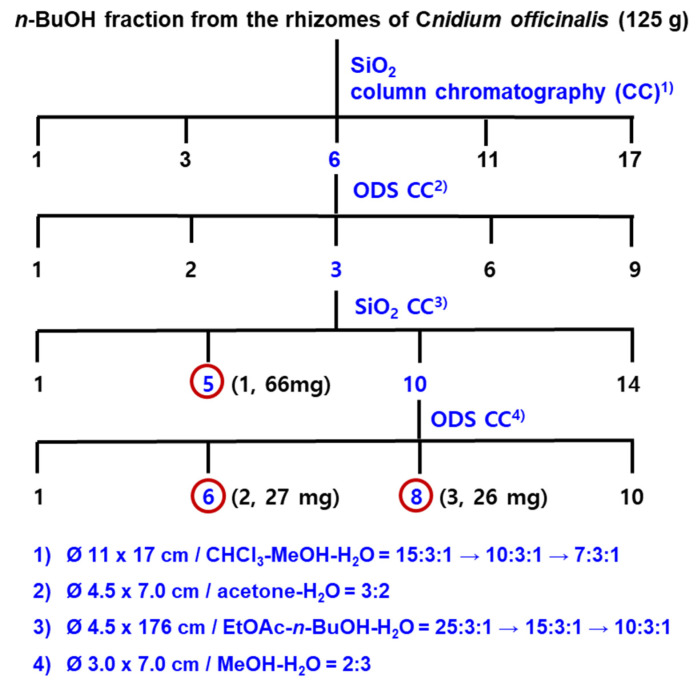
Isolation of phthalide glycosides from the *n*-BuOH fraction of *Cnidium officinale* rhizomes.

Ten kilograms of the dried rhizomes of *Cnidium officinale* Makino (10 kg) were soaked in aqueous ethanol (70% EtOH, 54 L × 2) at room temperature for 12 h. The concentrated brownish residue (2.1 kg) was poured into H_2_O (4 L) and extracted with EtOAc (4 L × 3) and *n*-BuOH (3.2 L × 3), successively, to yield EtOAc (COE, 280 g), *n*-BuOH (COB, 125 g), and H_2_O (COW, 1.695 kg) Fr. The CC for COB (120 g) was carried out using SiO_2_ resin (Figure 3) to give 17 fractions. The subsequent repeated CC using SiO_2_ and ODS as packing materials yielded three new phthalide glycosides, compounds 1–3 (Figure 4).

**Ligusticoside C (1)**

White powder; TLC (SiO_2_) *Rf* 0.28, EtOAc-*n*-BuOH-H_2_O (15:3:1), (ODS) *Rf* 0.31, MeOH–H_2_O (2:3); [α]_D_ −7.0 (c 0.01, CH_3_OH); IR (LiF plates) *ν*_max_ 3367, 2928, 1742, 1606, 1514, 1455 cm^−1^; CMR and PMR: Table 1 and Table 2. ESI-MS: *m*/*z* 373.1861 [M + H]^+^ (calcd for C_18_H_29_O_8_, 373.1858).

**Ligusticoside D (2)**

White powder; TLC (SiO_2_) *Rf* 0.44, CHCl_3_-MeOH-H_2_O (7:3:1), (ODS) *Rf* 0.30, MeOH–H_2_O (2:3); [*α*]_D_ −47.0 (*c* 0.01, CH_3_OH); IR (LiF plates) *ν*_max_ 3356, 2930, 1747, 1600, 1456 cm^−1^; CMR and PMR: Table 1 and Table 2; ESI-MS: *m*/*z* 505.2285 [M + H]^+^ (calcd for C_23_H_37_O_12_, 505.2281).

**Ligusticoside E (3)**

White powder; TLC (SiO_2_) *Rf* 0.48, CHCl_3_-MeOH-H_2_O (7:3:1), (ODS) *Rf* 0.27, MeOH–H_2_O (2:3); [*α*]_D_ −135.0 (*c* 0.01, CH_3_OH); IR (LiF plates) *ν*_max_ 3357, 2929, 1746, 1602, 1458 cm^−1^; CMR and PMR: Table 1 and Table 2; ESI-MS: *m*/*z* 505.2283 [M + H]^+^ (calcd for C_23_H_37_O_12_, 505.2281).

### 3.4. Acid Hydrolysis of Phthalide Glycosides ***1**–**3***

A quantity of 10 mg of each glycoside was dissolved in 1N HCl (5 mL) and refluxed for 3 hrs. H_2_O (15 mL) was added to the reaction mixture followed by being extracted with EtOAc (20 mL × 2). The EtOAc phase was concentrated in vacuo and purified through open SiO_2_ CC (2 × 8 cm) using CHCl_3_–MeOH (10:1) to give each aglycone (**1a**, 3 mg; **2a**, 4 mg; **3a**, 3 mg).

**1a**: Colorless oil; CD (CH_3_OH) λ nm (Δε) 255 (3.22), 212 (–3.26); PMR (600 MHz, CDCl_3_, δ_H_, coupling pattern, *J* in Hz) 5.84 (1H, br. dd, 1.8, 10.2, H-6), 5.73–5.70 (1H, m, H-7), 4.48 (1H, ddd, 4.4, 4.8, 9.6, H-3), 3.84 (1H, ddd, 3.6, 4.8, 7.4, H-4), 3.34–3.31 (1H, m, H-8), 2.49 (1H, m, H-5a), 2.28 (1H, ddd, 4.9, 4.3, 9.5, H-9), 2.16–2.13 (1H, m, H-10a), 1.97 (1H, overlapped, H-10b), 1.95 (1H, overlapped, H-5b), 1.33–1.28 (2H, overlapped, H-11), 1.21 (2H, overlapped, H-12), 0.81 (3H, t, 7.8, H-13).

**2a**: Colorless oil; CD (CH_3_OH) λ nm (Δε) 250 (–1.66), 218 (1.29); PMR (600 MHz, DMSO-*d*_6_, δ_H_, coupling pattern, *J* in Hz) 5.75 (1H, ddd, 1.2, 6.0, 6.0, H-6), 5.71 (1H, dd, 9.6, 3.0, H-7), 4.48 (1H, ddd, 5.4, 5.4, 8.4, H-3), 3.93 (1H, ddd, 5.4, 8.4, 10.8, H-4), 3.61–3.59 (1H, m, H-8), 2.61, (1H, ddd, 5.4, 5.4, 10.8, H-5a), 2.59–2.56 (1H, m, H-9), 2.00–1.96 (1H, m, H-10a), 1.91–1.87 (1H, m, H-10b), 1.85–1.81 (1H, m, H-5b), 1.41–1.35 (2H, m, H-11), 1.34–1.29 (2H, m, H-12), 0.88 (3H, t, 6.9, H-13).

**3a**: Colorless oil; CD (CH_3_OH) λ nm (Δε) 257 (2.99), 214 (–2.63); PMR: same as **1a**.

### 3.5. Evaluation of the Recovery Effect on Otic Hair Cells in Zebrafish Damaged by Neomycin Treatment 

The evaluation of the recovery effect was followed by the methods previously reported in the literature [15].

## 4. Conclusions

Column chromatography for an active Fr, the *n*-BuOH Fr obtained from *Cnidium officinale* rhizomes, yielded three new phthalide glycosides, named ligusticosides C (**1**), D (**2**), and E (**3**). As each aglycone had four chiral carbons, their relative and absolute stereostructures were determined by examining their NOESY and circular dichroism (CD) data. This is the first report of the isolation of phthalide glycosides to have double bonds at C-6 and C-7. Ligusticoside C (**1**) and ligusticoside E (**3**) (0.1 μM) recovered the damage (*p* < 0.01) caused by neomycin treatment in zebrafish otic hair cells.

## Figures and Tables

**Table 1:** ^13^C-NMR data on the phthalide glycosides from the rhizome of *Cnidium officinale* Makino (125 MHz, CD_3_OD, δ_C_).

No. of C	Phthalide Glycosides *		
	1	2	3
1	179.04	179.12	179.29
3	83.20	84.77	83.40
4	73.19	68.62	73.99
5	27.75	29.87	28.10
6	127.66	127.42	127.86
7	122.29	122.83	122.51
8	42.67	45.06	42.96
9	43.90	44.33	44.07
10	36.06	31.66	36.26
11	28.77	30.23	29.00
12	23.51	23.87	23.74
13	14.31	14.63	14.54
Glc **-1′	102.76	99.98	103.41
2′	75.03	75.41	75.15
3′	78.01	78.18	78.13
4′	71.85	72.25	72.01
5′	78.05	77.24	77.25
6′	63.08	69.60	69.19
Api ***-1′′	-	111.38	111.19
2′′	-	78.13	78.16
3′′	-	80.52	80.62
4′′	-	74.96	75.09
5′′	-	65.68	65.57

* **1**, ligusticoside C; **2**, ligusticoside D; **3**, ligusticoside E. ** Glc, *β*-D-glucopyranosyl. *** Api, *β*-D-apiofuranosyl.

**Table 2:** ^1^H-NMR data on the phthalide glycosides from the rhizome of *Cnidium officinale* Makino (600 MHz, CD_3_OD, δ_H_, coupling pattern, *J* in Hz).

No. of H	Phthalide Glycosides *		
	1	2	3
3	4.55, ddd, 5.4, 5.4, 9.6	4.57, ddd, 5.4, 5.4, 8.4	4.65, ddd, 4.2, 4.8, 9.6
4	4.12, ddd, 3.6, 4.8, 6.6	4.15, ddd, 5.4, 8.4, 10.2	4.17, ddd, 4.8, 4.8, 6.6
5	2.21, overlapped 2.19, overlapped	2.71, ddd, 5.4, 5.4, 10.2 2.02, overlapped	2.32, overlapped 2.30, overlapped
6	5.72, br.ddd, 4.2, 6.6, 10.2	5.85, overlapped	5.83, br. ddd, 2.4, 4.2, 9.6
7	5.71, ddd, 2.4, 4.2,10.2	5.84, overlapped	5.72, dddd, 1.8, 1.8, 4.2, 9.6
8	3.27, overlapped	3.60, overlapped	3.42, overlapped
9	2.71, ddd, 4.8, 4.2, 9.6	2.78–2.74, m	2.85, ddd, 4.8, 4.2, 9.6
10	1.83–1.77, m 1.60–1.54, m	2.18–2.12, m 2.05, overlapped	1.96–1.92, m 1.71–1.63, m
11	1.46–1.39, m 1.27–1.21, overlapped	1.54–1.48, m	1.50–1.48, m 1.36, overlapped
12	1.27, overlapped	1.50–1.41, m	1.39, overlapped
13	0.84, t, 7.2	1.00, t, 7.2	0.95, t, 7.2
Glc **-1′	4.22, d, 7.8	4.52, d, 7.8	4.31, d, 7.8
2′	3.01, dd, 7.8, 9.0	3.20, dd, 7.8, 9.3	3.11, dd, 7.8, 8.4
3′	3.15, dd, 9.0, 9.0	3.37, dd, 9.3, 9.3	3.32, dd, 9.3, 8.4
4′	3.24, dd, 9.0, 9.0	3.24, dd, 9.3, 9.3	3.23, dd, 9.3, 9.3
5′	3.30, m	3.45, ddd, 1.8, 6.6, 9.3	3.40, ddd, 1.8, 7.2, 9.3
6′	3.79, dd, 2.8, 12.0 3.53, dd, 4.2, 12.0	4.04, dd, 1.8, 11.4 3.57, dd, 6.6, 11.4	3.99, dd, 1.8, 11.4 3.61, dd, 7.2, 11.4
Api ***-1′′	-	5.05, d, 3.0	5.04, d, 2.4
2′′	-	3.90, d, 3.3	3.89, d, 2.4
3′′	-	-	-
4′′	-	3.98, d, 9.6 3.79, d, 9.6	3.96, d, 9.6 3.76, d, 9.6
5′′	-	3.58, s	3.57, s

* **1**, ligusticoside C; **2**, ligusticoside D; **3**, ligusticoside E. ** Glc, *β*-D-glucopyranosyl. *** Api, *β*-D-apiofuranosyl.

## Data Availability

The data presented in this study are available in the article and Supplementary Materials.

## Supplementary Materials

The following are available online, Figures S1–S3: ^13^C and ^1^H NMR spectra of ligusticosides C, D, and E. Figures S4–S6: 2-D NMR spectra of ligusticosides C, D, and E.
