# Supplementary material for: Three New Phthalide Glycosides from the Rhizomes of Cnidium officinale and Their Recovery Effect on Damaged Otic Hair Cells in Zebrafish
Source: Molecules. 2021 Nov 21;26(22):7034. doi: 10.3390/molecules26227034 (PMC8624857; doi:10.3390/molecules26227034)
Supplement: Supplementary file 1 [file molecules-26-07034-s001.zip › molecules-1462248-supplementary.pdf]

Supplementary Materials

Three new phthalide glycosides from the rhizomes of *Cnidium officinalis* and their recovery effect on damaged otic hair cells in zebrafish

Hyoung-Geun Kim [1†], Seon Min Oh [1, 2†], Na Woo Kim [1], Ji Heon Shim [1], Youn Hee Nam [1], Nguyen Trong Nguyen [1], Min-Ho Lee [3], Dae Young Lee [2], Tong Ho Kang [1], Nam-In Baek [1,*]

[1] Graduate School of Biotechnology and Department of Oriental Medicinal Biotechnology, Kyung Hee University, Yongin 17104, Korea; zwang05@khu.ac.kr (H.-G.K.); seonmin88@khu.ac.kr (S.M.O.); nawoonifty@khu.ac.kr (N.W.K.); jee1015235@gmail.com (J.H.S.); 01030084217@hanmail.net (Y.H.N.); ntnguyen@khu.ac.kr (N.T.N.); panjae@khu.ac.kr (T.H.K.)

[2] Department of Herbal Crop Research, National Institute of Horticultural and Herbal Science, RDA, Eumseong 27709, Republic of Korea; seonmin88@khu.ac.kr (S.M.O.); dylee0809@gmail.com (D.Y.L.)

[3] Department of Food Technology and Services, Eulji University, Seongnam 13135, Republic of Korea; minho@eulji.ac.kr (M.-H.L.)

* Correspondence: nibaek@khu.ac.kr; Tel.: +82-31-201-2661

† Co-first author, these authors contributed equally to this work

Contents:

S1: ^{13}C and ^{1}H NMR spectra of ligusticoside C (600 MHz, CD_3OD).

S2: ^{13}C and ^{1}H NMR spectra of ligusticoside D (600 MHz, CD_3OD).

S3: ^{13}C and ^{1}H NMR spectra of ligusticoside E (600 MHz, CD_3OD).

S4. ^{1}H-^{1}HCOSY, HSQC, and HMBC spectra of ligusticoside C (600 MHz, CD_3OD).

S5. ^{1}H-^{1}HCOSY, HSQC, and HMBC spectra of ligusticoside D (600 MHz, CD_3OD).

S6. ^{1}H-^{1}HCOSY, HSQC, and HMBC spectra of ligusticoside E (600 MHz, CD_3OD).

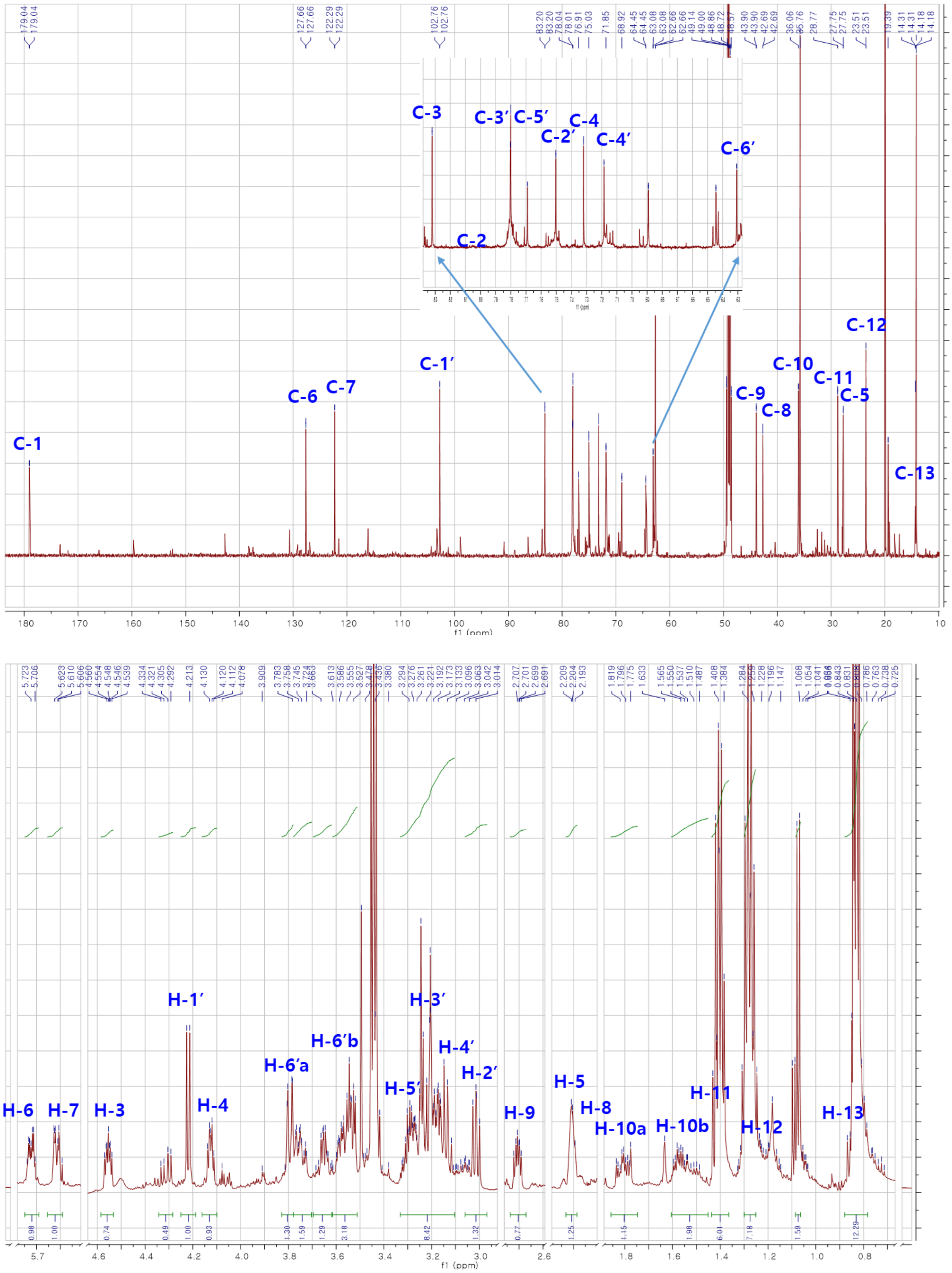

Figure S1. ^{13}C and ^{1}H NMR spectra of ligusticoside C (600 MHz, CD_3OD).

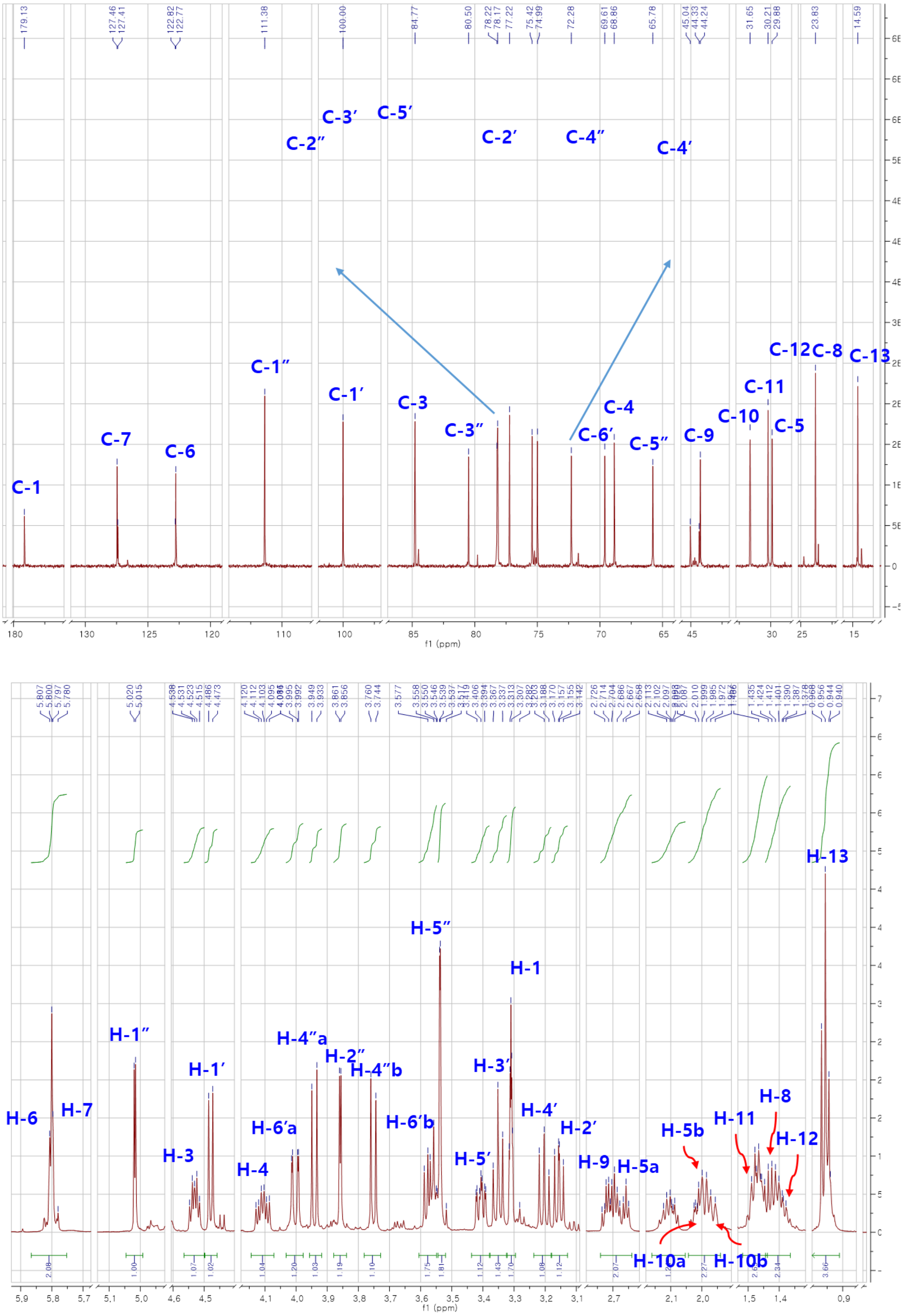

Figure S2. ^{13}C and ^{1}H NMR spectra of ligusticoside D (600 MHz, CD_3OD).

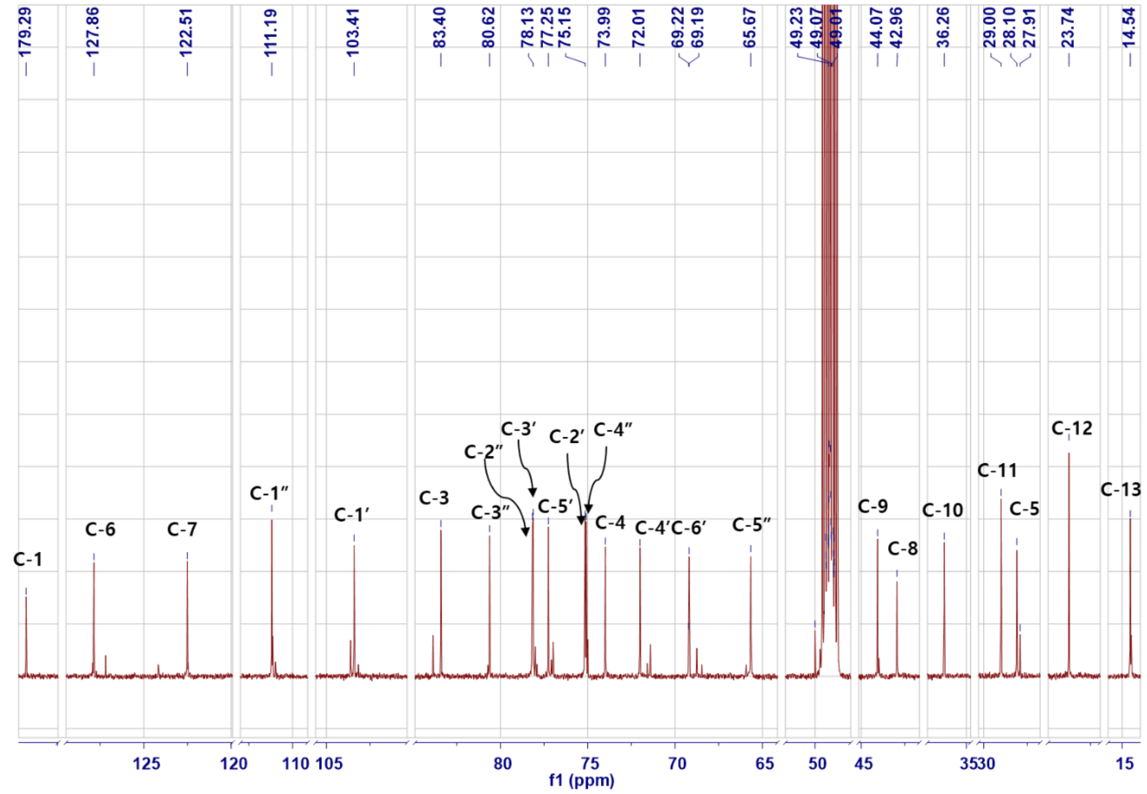

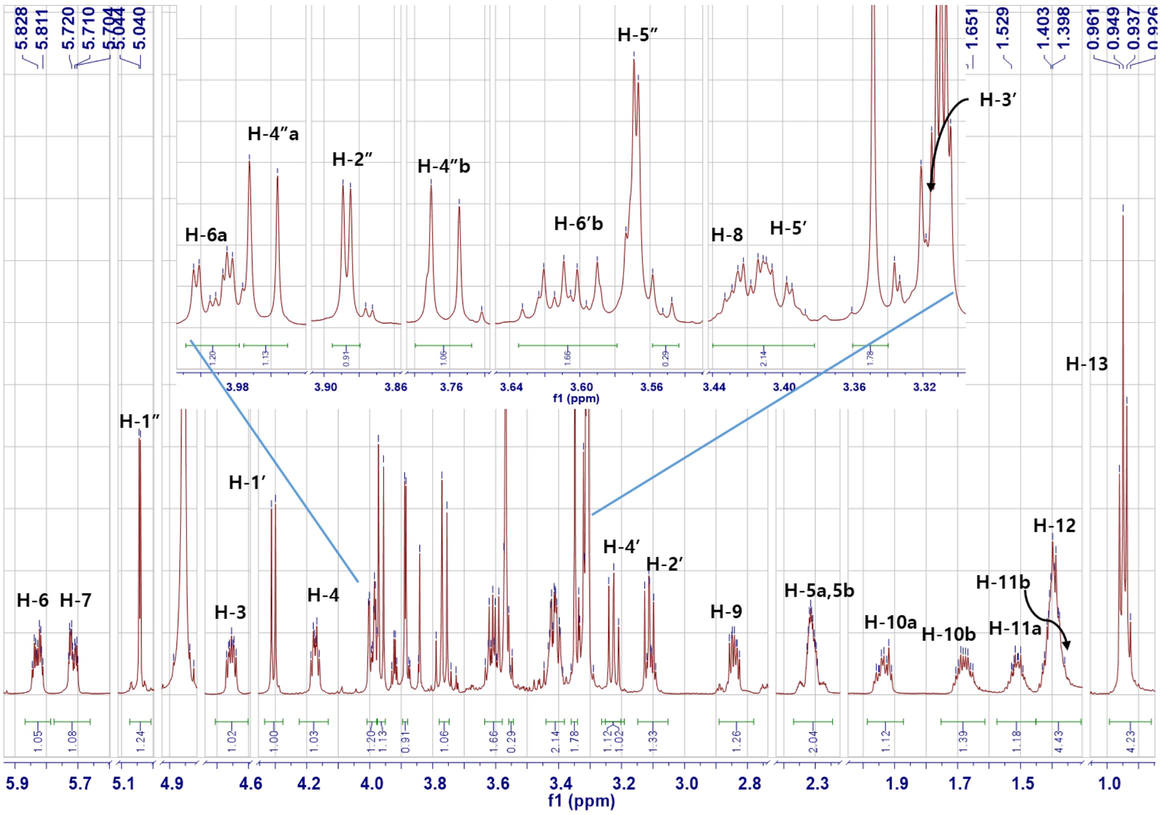

Figure S3. ^{13}C and ^{1}H NMR spectra of ligusticoside E (600 MHz, CD_3OD).

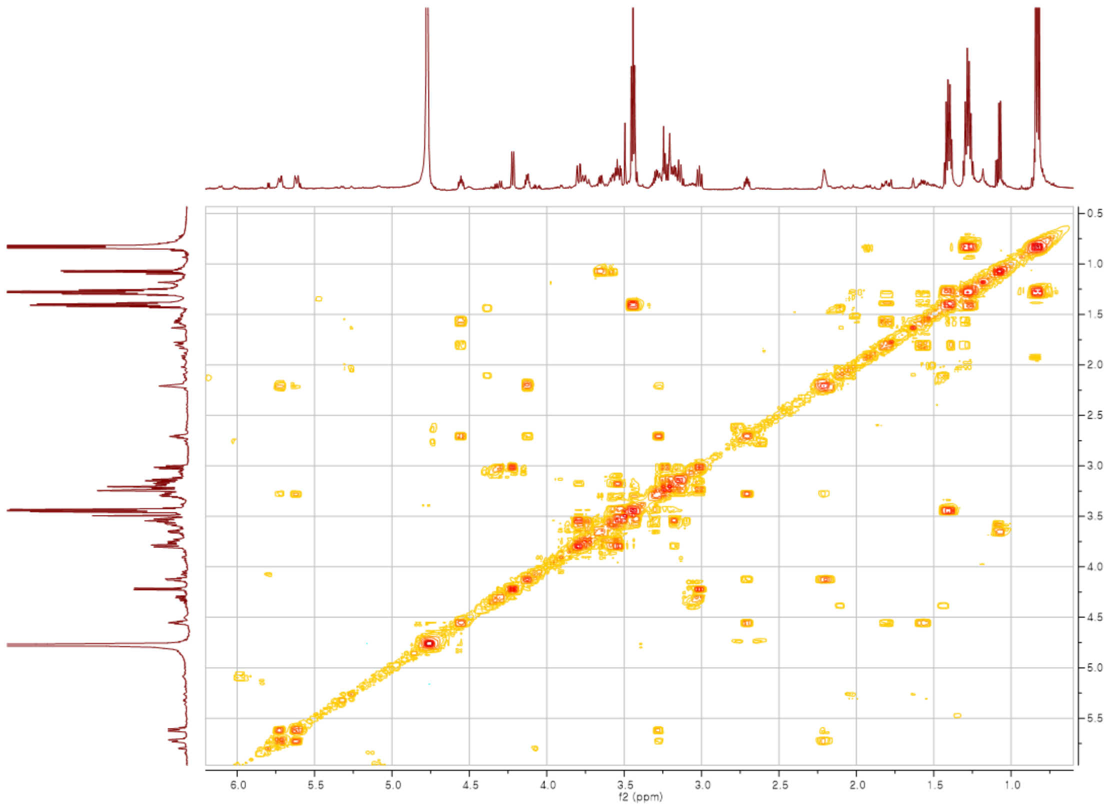
6.0
5.5
5.0
4.5
4.0
3.5
3.0
2.5
2.0
1.5
1.0
f2 (ppm)
0.5
1.0
1.5
2.0
2.5
3.0
3.5
4.0
4.5
5.0
5.5

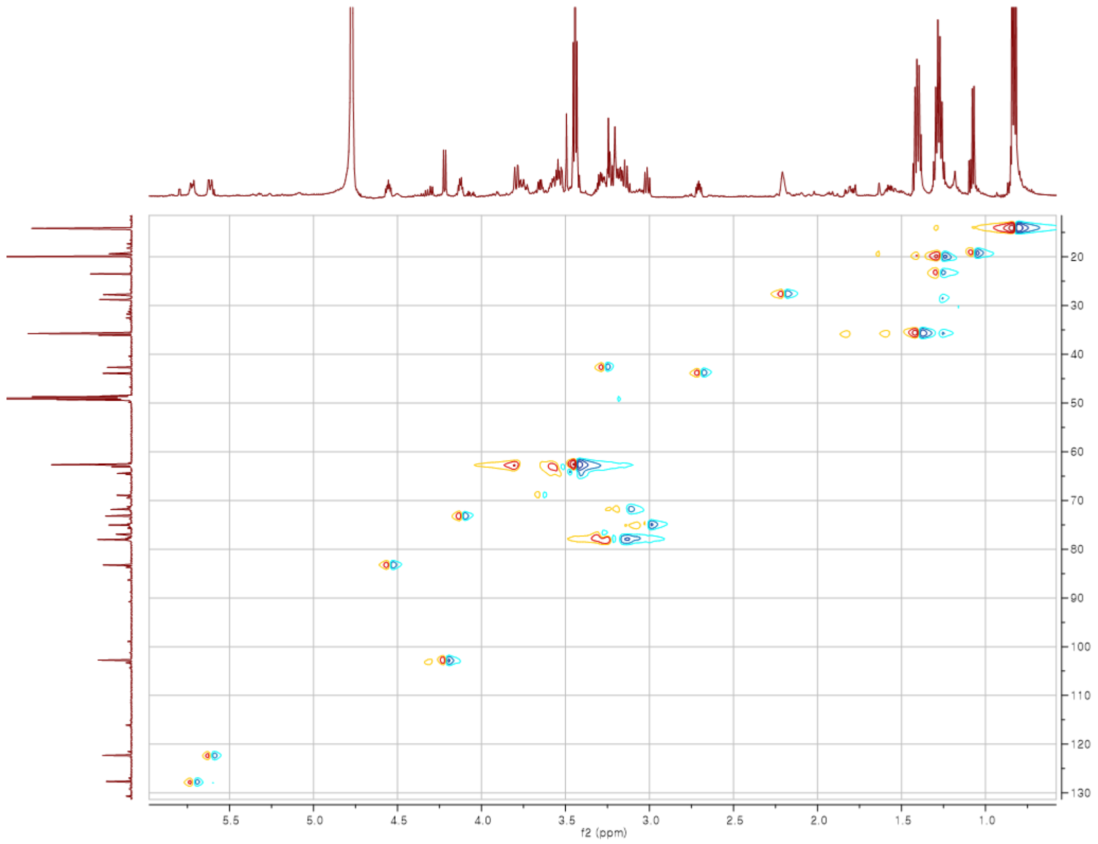
5.5
5.0
4.5
4.0
3.5
3.0
2.5
2.0
1.5
1.0
f2 (ppm)
20
30
40
50
60
70
80
90
100
110
120
130

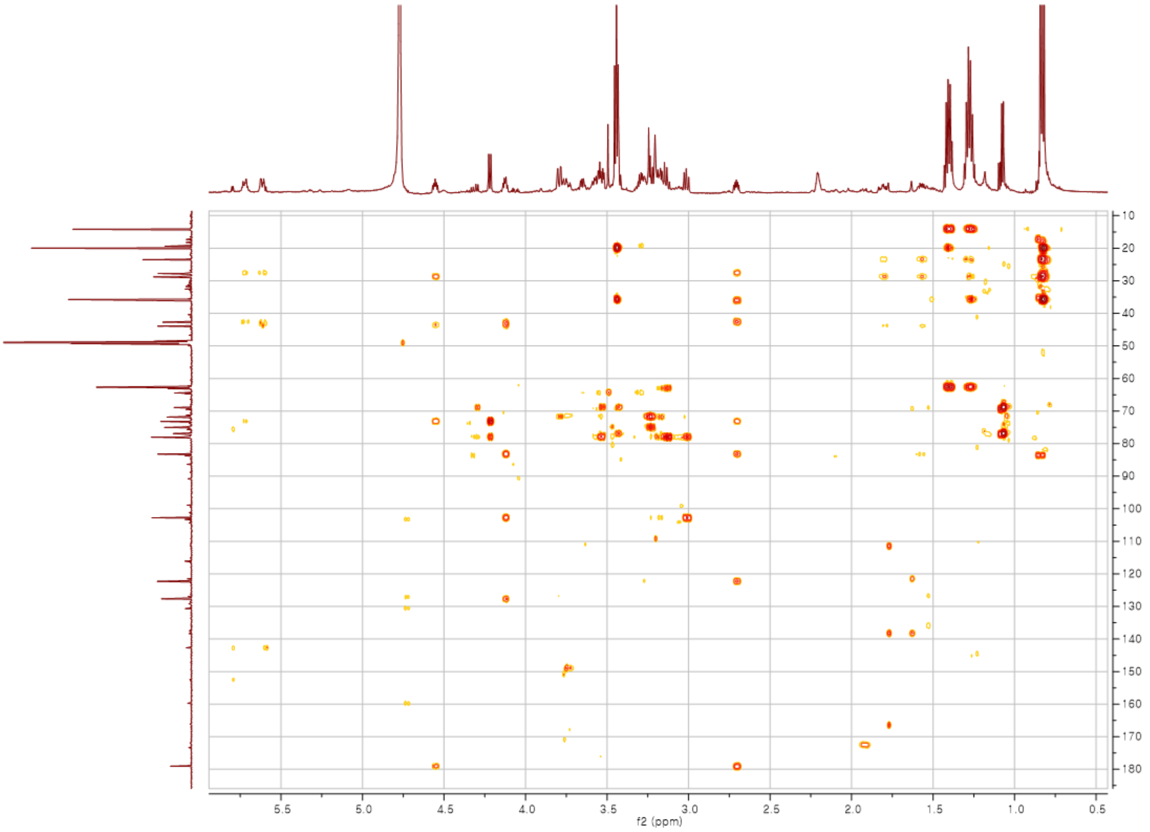

Figure S4. ^{1}H-^{1}H COSY, HSQC,and HMBC spectra of ligusticoside C (600 MHz, CD_3OD).

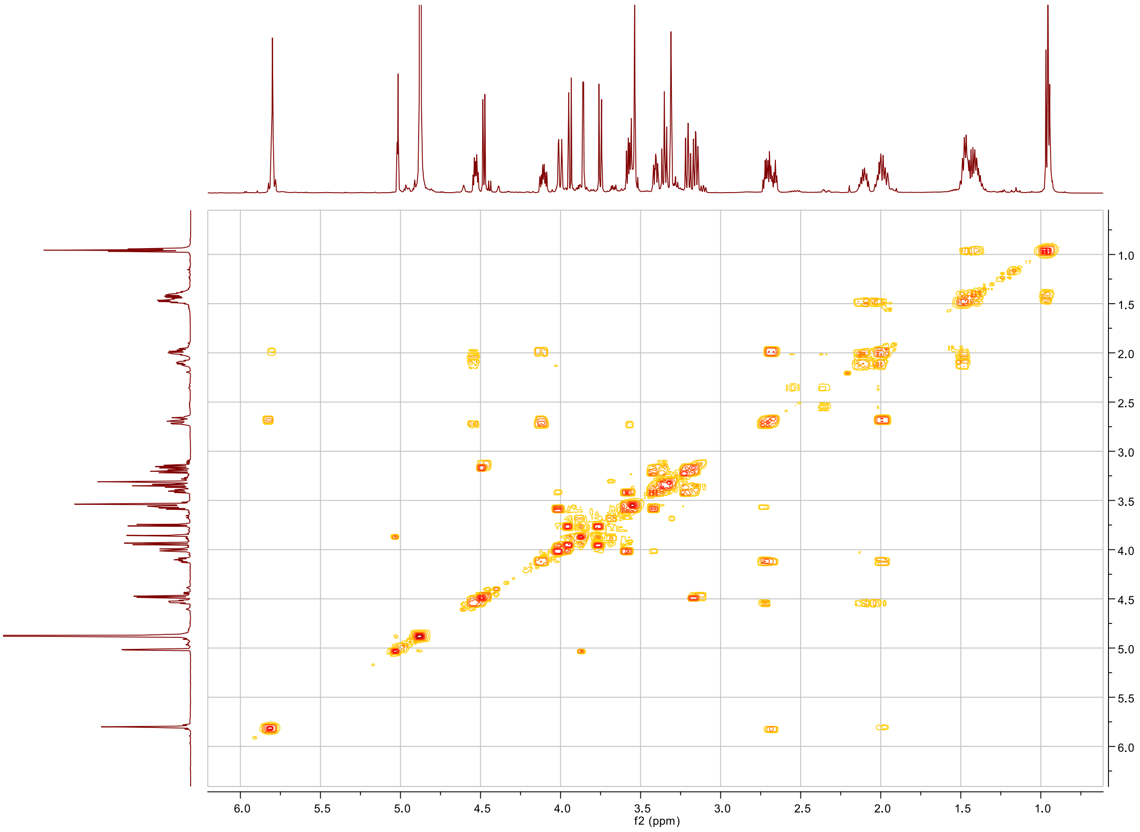
6.0
5.5
5.0
4.5
4.0
3.5
3.0
2.5
2.0
1.5
1.0
f2 (ppm)

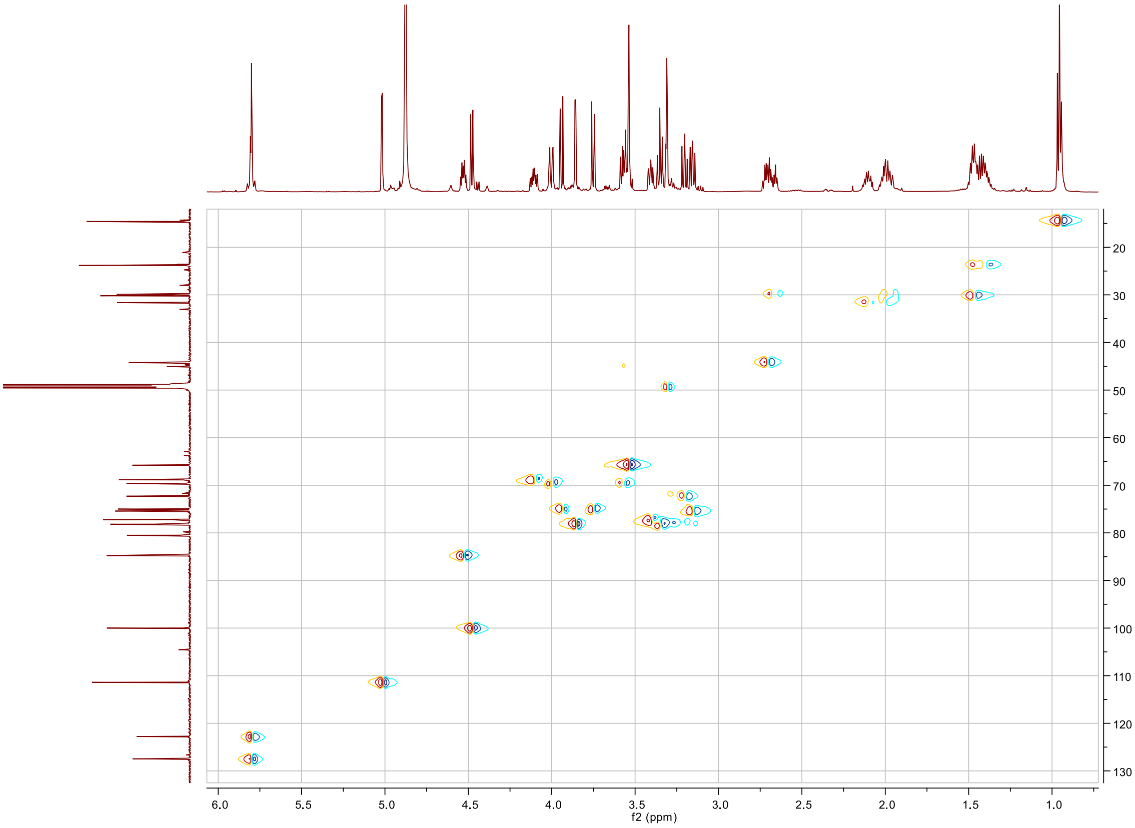
20
30
40
50
60
70
80
90
100
110
120
130
6.0
5.5
5.0
4.5
4.0
3.5
3.0
2.5
2.0
1.5
1.0
f2 (ppm)

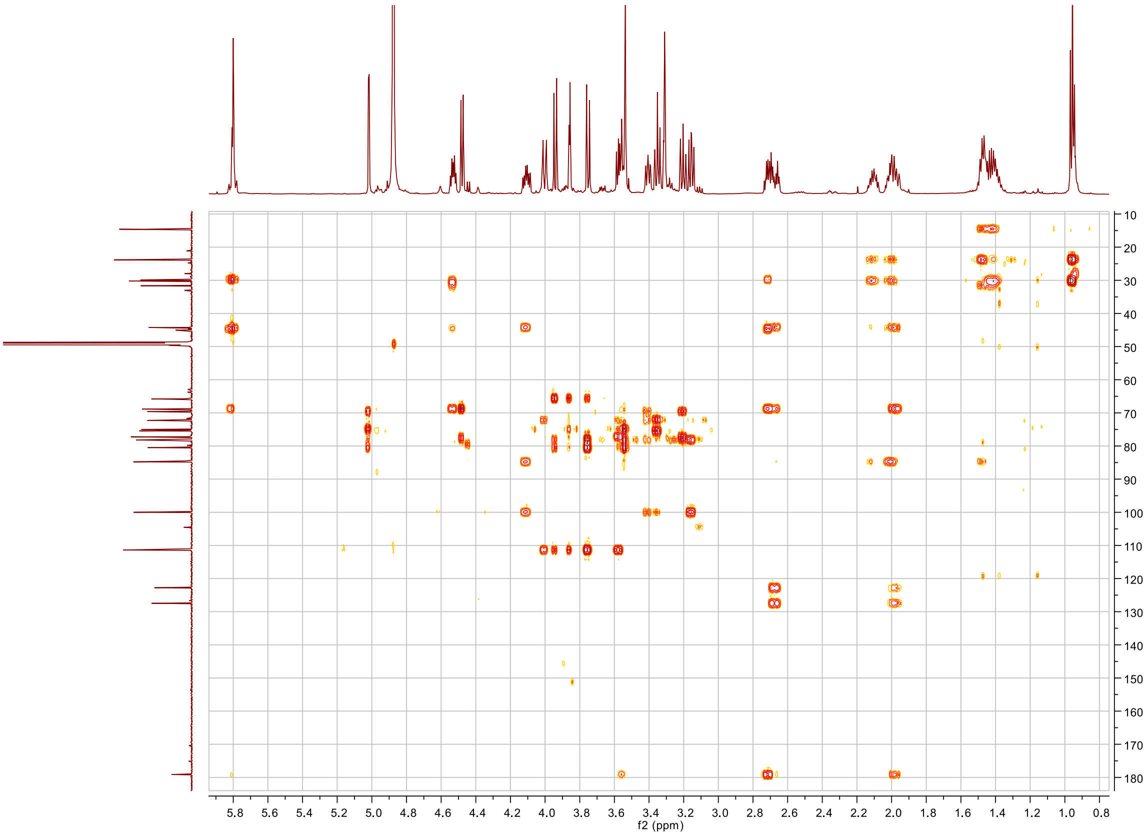

Figure S5. ^{1}H-^{1}H COSY, HSQC,and HMBC spectra of ligusticoside D (600 MHz, CD_3OD).

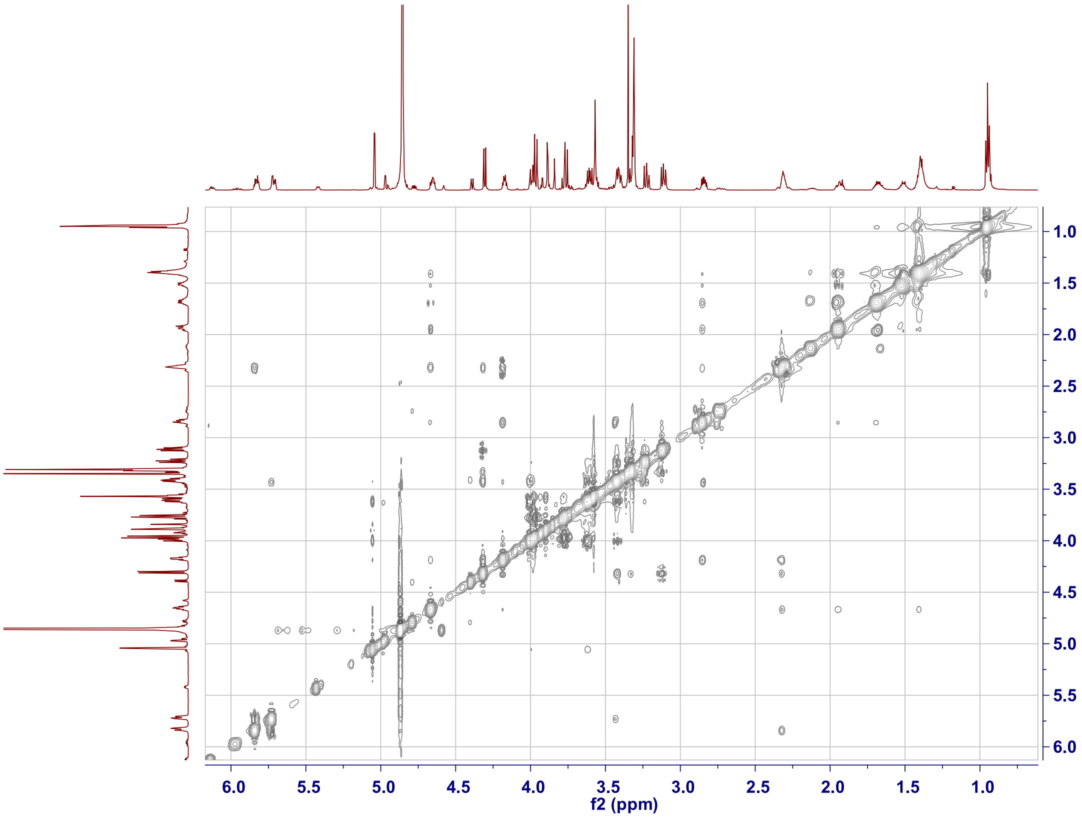
f2 (ppm)

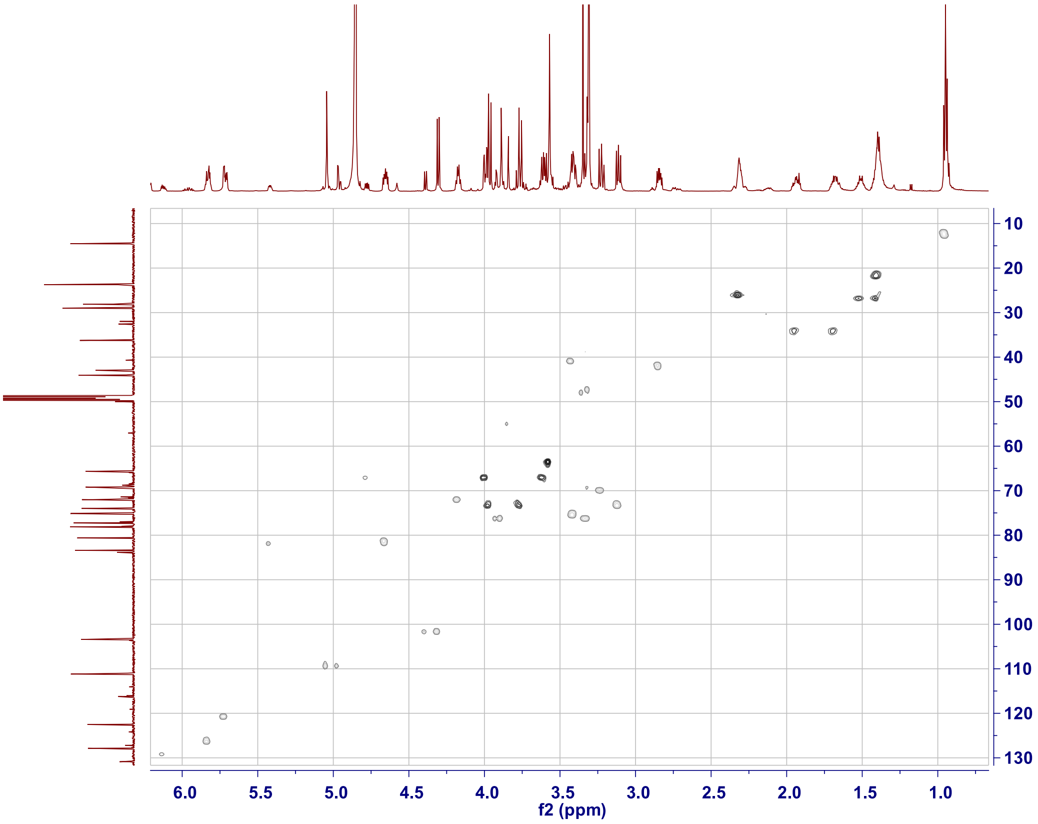
f2 (ppm)

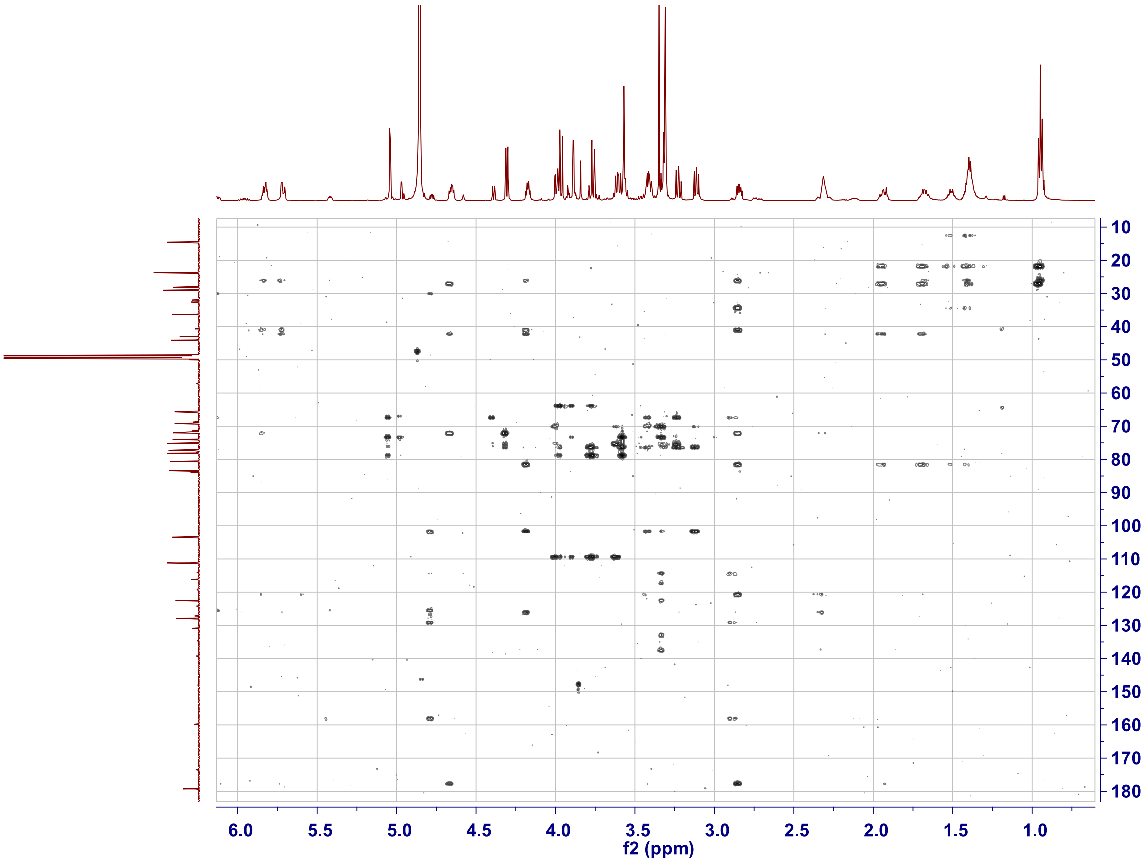

Figure S6. ^{1}H-^{1}H COSY, HSQC,and HMBC spectra of ligusticoside D (600 MHz, CD_3OD).
